# Fatty acid synthase, a novel poor prognostic factor for acute lymphoblastic leukemia which can be targeted by ginger extract

**DOI:** 10.1038/s41598-020-70839-9

**Published:** 2020-08-21

**Authors:** Maryam Harouni Ghaeidamini, Soheila Rahgozar, Somayeh Babasheikhali Rahimi, Arman Safavi, Elaheh Sadat Ghodousi

**Affiliations:** Department of Cell and Molecular Biology and Microbiology, Faculty of Biological Science and Technology, University of Isfahan, 81746-73441 Isfahan, Iran

**Keywords:** Cancer, Haematological cancer, Leukaemia, Acute lymphocytic leukaemia

## Abstract

Altered metabolism of fatty acid synthesis is considered a hallmark characteristic of several malignancies, including acute lymphoblastic leukemia (ALL). To evaluate the impact of fatty acid synthase (FASN) on drug resistant ALL, bone marrow samples were collected from 65 pediatric ALLs, including 40 de novo and 25 relapsed patients. 22 non-cancer individuals were chosen as controls. Quantitative RT-PCR showed increased expression levels of *FASN* in drug resistant patients compared with the therapy responders. Single and combined treatment of malignant cells were analyzed using Annexin-V/PI double staining and MTT assays. Incubation of resistant primary cells with ginger showed simultaneous increased apoptosis rates and reduced *FASN* expression levels. Furthermore, docking studies demonstrated high affinity bindings between ginger derivatives and FASN thioesterase and ketosynthase domains, compared with their known inhibitors, fenofibrate and morin, respectively. Finally, combined treatment of in-house multidrug resistant T-ALL subline with ginger and dexamethasone induced drug sensitivity and down regulation of *FASN* expression, accordingly. To the best of our knowledge, this is the first study that introduces *FASN* upregulation as a poor prognostic factor for drug resistant childhood ALL. Moreover, it was revealed that FASN inhibition may be applied by ginger phytochemicals and overcome dexamethasone resistance, subsequently.

## Introduction

Acute lymphoblastic leukemia (ALL) is the most common type of hematological malignancy in children^1,2^. Despite the enormous advances in modern medicine and development of innovative therapeutic strategies, disease relapse remains a leading cause of cancer-related morbidity and mortality in children^3^. Metabolic rearrangements are vital to satisfy the different requirements of cancer cells during tumorigenesis^4^. Elevated de novo fatty acid biosynthesis is a hallmark adaptation in many cancers that supply signaling molecules and basic elements for lipid biosynthesis^5^. While most normal cells supply their fatty acids from dietary sources, cancer cells reactivate de novo fatty acid synthesis^6^. Fatty acid synthase (FASN) is a multifunctional protein containing six enzymatic domains that catalyzes the biosynthesis of palmitate^5^. Elevated expression of *FASN* is found to be associated with poor prognosis and higher risk of recurrence in a number of human cancers. Indeed, *FASN* overexpression has been shown to contribute to multidrug resistance (MDR). Multi-drug resistance is one of the major obstacles to the successful treatment of various types of cancer, particularly childhood ALL^5,7,8^.

Glucocorticoids (GCs) such as prednisone and dexamethasone (DEX) are indispensable drugs for childhood ALL treatment^9^. Early response to glucocorticoids is a positive prognostic indicator and glucocorticoid resistance has been associated with an increased risk of relapse and poor clinical response^10,11^. Glucocorticoids regulate *FASN* expression and subsequently affect lipogenesis^12^. Therefore, FASN knock down or inhibition of its activity is recognized as an attractive therapeutic approach. Moreover, FASN can be considered as a target in combinational therapy. However, early generation of FASN inhibitors including cerulenin, orlistat and C75 have limitations such as chemical instability, low bioavailability and undesirable side effects like body weight loss, that restrict their clinical development^5,13^.

The aim of the current study was to evaluate *FASN* expression levels in children with ALL and those who were resistant to chemotherapy. Furthermore, we examined the effect of ginger extract (*Zingiber officinale*) on *FASN* expression levels in leukemic cell lines and patients primary cells. Recent studies revealed that some ginger components can reduce FASN expression. Therefore, combined treatment was performed with ginger and dexamethasone together on the CCRF-CEM/MVCD resistant subline. In the final step, molecular docking was recruited to determine the best ginger phytochemicals which could interfere with FASN activity through binding its first and last catalytic domains. To the best of our knowledge, this is the first study to introduce *FASN* as a poor prognostic marker for pediatric ALL patients. In addition, we evaluated the cytotoxicity of ginger extract and its capacity to down-regulate *FASN* expression in ALL relapsed patients. Finally, we demonstrated that ginger phytochemicals may inhibit FASN activity and ginger may induce susceptibility to dexamethasone in the ALL resistant subline, and decrease *FASN* expression levels, accordingly.

## Results

### Patients characteristics

The clinical characteristics of the ALL patients are summarized in Table 1b. Among the 65 patients, 40 cases were newly diagnosed, and 25 patients were relapsed cases. 22 non-cancer bone marrow specimens were used as the control group. Controls were age/gender-matched children (12 (54.5%) males and 10 (45.5%) females < 12 years of age) who were administered to the hospital with thrombocytopenia. However, no evidence of cancer was found in their bone marrow aspirates. Regarding their response to one year chemotherapy, the newly diagnosed patients were divided into 9 MRD+ and 31 MRD− patients followed by PCR-SSCP analyses.

**Table 1:** (a) Ginger extract components (according to the manufacturer), (b) patient characteristics.

(a)		
Analysed ingredients	Method of examination	
Gingerols (3%)	HPLC	
Heavy metals (NMT 10 ppm)	USP231	
Arsenic (NMT 0.5 ppm)	ICP-MS	
Mercury (NMT 0.05 ppm)	ICP-MS	
Lead (NMT 0.2 ppm)	ICP-MS	
Aflatoxin (NMT 8 ppb)	HPLC	

(b)		
Patient characteristics	Number of patients N = 65	%
Sex		
Male	35	53.8
Female	30	46.1
ALL immunophenotype		
T cell linage	6	9.23
Pre-B and early pre-B linage	59	90.76
Cytogenetics		
T (4;11)/KMT2A-AFF1	4	6.15
T (9;22)/BCR-ABL1	2	3.07
T(1;19)/TCF3-PBX1	0	0
T(12;21)/ETV6-RUNX1	0	0
MRD−		
Pre-B cell	29	44.61
T cell	2	3.07
Total MRD−	31	47.69
MRD+		
Pre-B cell	7	10.76
T cell	2	3.07
Total MRD+	9	13.84
Relapse		
Pre-B cell	23	35.38
T cell	2	3.07
Total relapse	25	38.46

*USP* United States Pharmacopeia, *NMT* not more than, *ppm* parts per million, *ppb* parts per billion, *HPLC* high-performance liquid chromatography, *ICP-MS* inductively coupled plasma mass spectrometry, *MRD* minimal residual disease in newly diagnosed patients, determined 1 year after the onset of treatment.

### Relative expression levels of *FASN* in de novo patients

**Figure 1:**
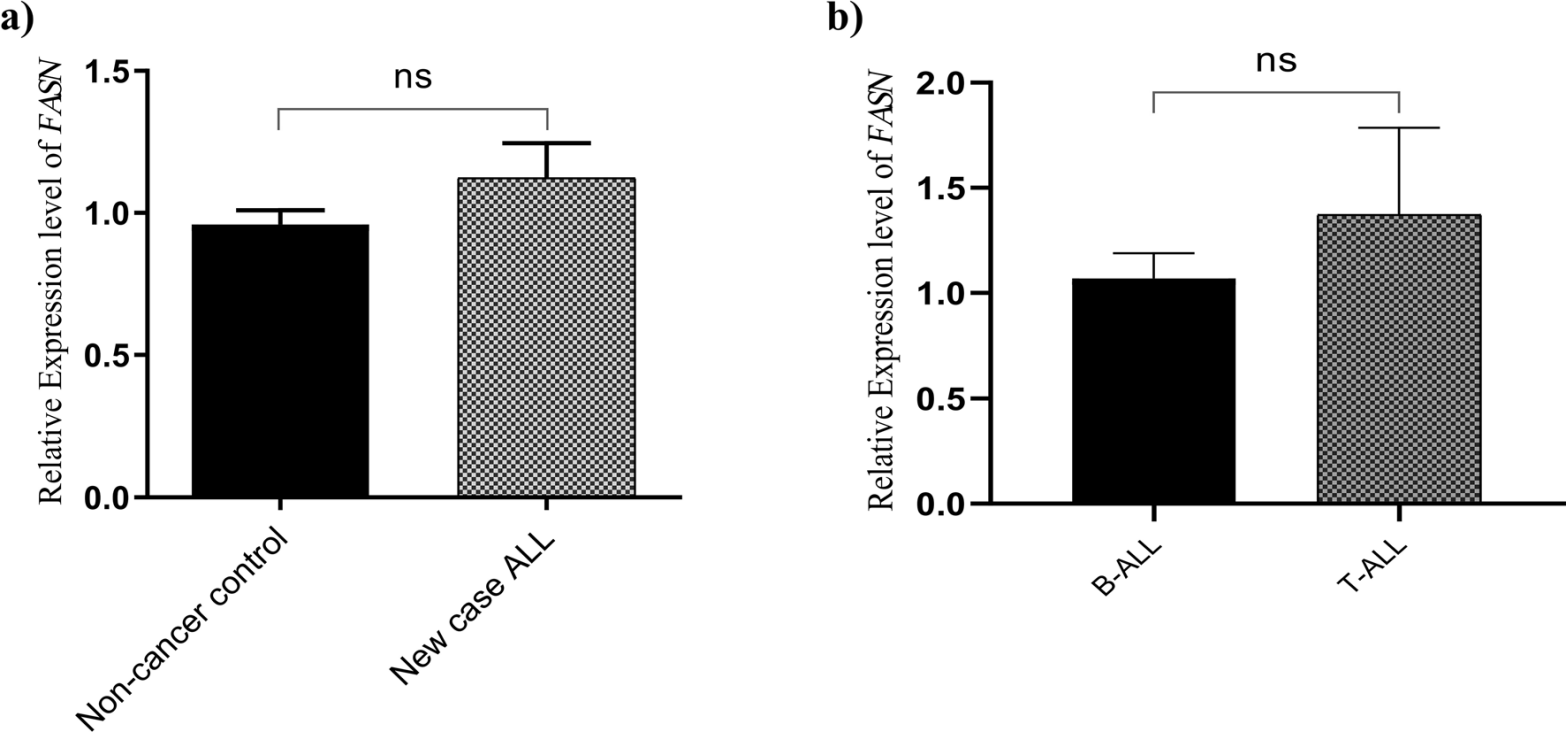
Expression levels of *FASN* in de novo ALL patient samples. (**a**) No significant variation in *FASN* expression was observed among de novo patients (n = 40) and non-cancer controls (n = 22) (*P* = 0.3432). The relative mRNA expression levels of *FASN* were normalized using *GAPDH* as an internal control. (**b**) Comparison of *FASN* mRNA expression levels showed no marked difference between B-ALL (n = 36) and T-ALL (n = 4) groups (*P* = 0.3432). Figures were created using GraphPad Prism8.0.2 (https://www.graphpad.com/). Results are presented as mean ± SEM. *ns *not significant.

In order to characterize the expression pattern of *FASN* in children with ALL, quantitative reverse transcriptase polymerase chain reaction was used to determine the expression levels of this gene in the bone marrow mononuclear cell samples of 40 children with newly diagnosed ALL and 22 non-cancer control cases. Results indicated no significant difference in the expression levels of *FASN* in de novo patients compared to the control group [1.123 ± 0.1228 vs. 0.9596 ± 0.05020 vs. mean ± SEM, *P* = 0.3432] (Fig. 1a).

To determine whether there was any significant difference between two subtypes of ALL, the mRNA expression levels of *FASN* was analyzed in 36 B-ALL and 4 T-ALL samples. As shown in Fig. 1b, there was no significant difference in *FASN* expression levels in these two groups [1.069 ± 0.1197 vs. 1.372 ± 0.4133, mean ± SEM, *P* = 0.3884].

### *FASN* expression levels in drug sensitive vs. resistant patients

**Figure 2:**
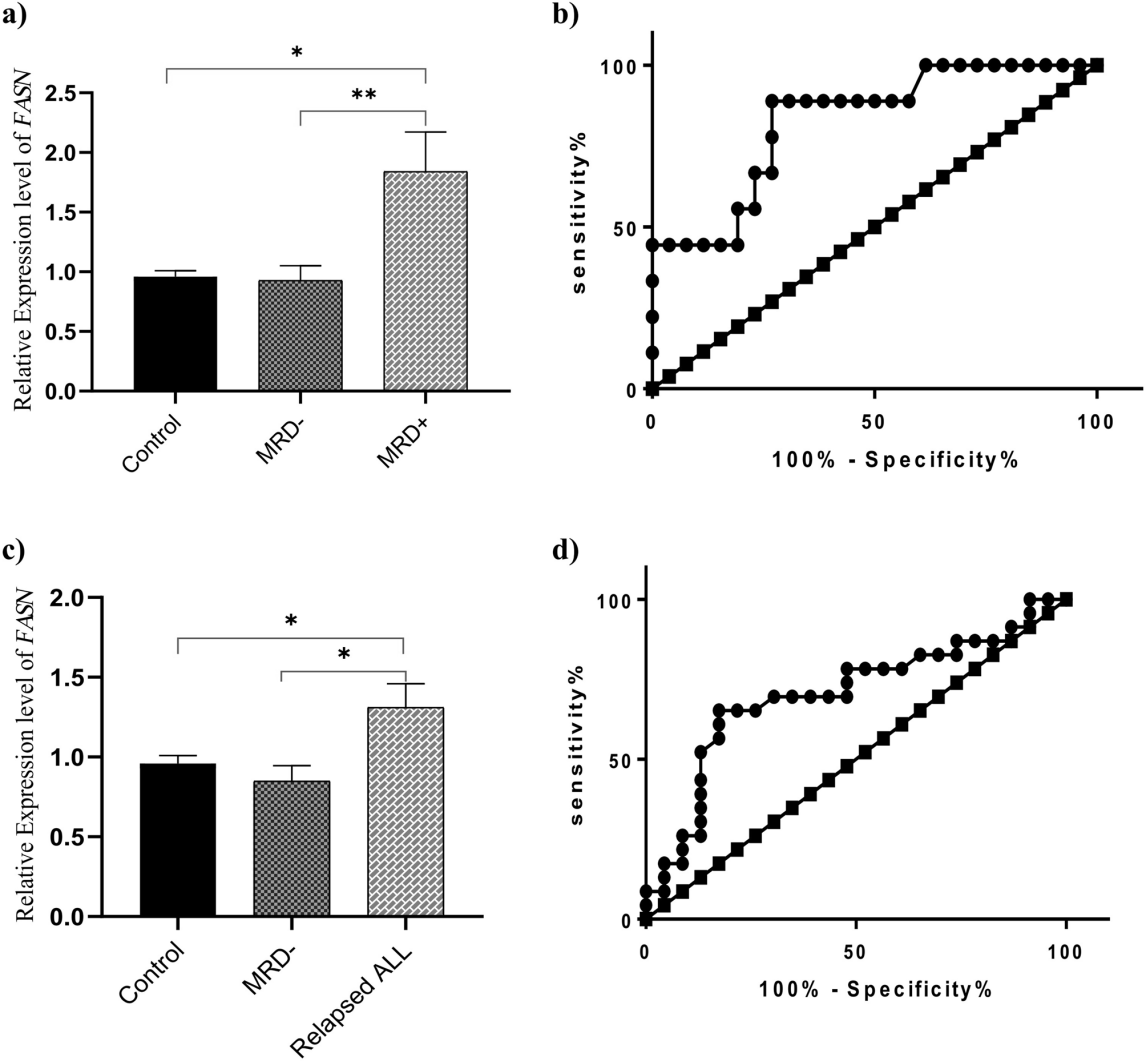
mRNA Expression levels of *FASN* in drug sensitive and resistant ALL patients. (**a**) *FASN* was overexpressed in the MRD+ patients (n = 9) in comparison with the MRD− individuals (n = 31) (*P* = 0.0021). (**b**) ROC analysis showed a prognostic potential of *FASN* to discriminate the resistant and sensitive patients to chemotherapeutic agents with 88.9% sensitivity and 73.1% specificity. (AUC = 0.8269, *P* = 0.0039). (**c**) *FASN* mRNA expression levels in relapsed group of ALL patients were significantly higher than those in the MRD− ones (*P* = 0.0180). (**d**) ROC analysis revealed that *FASN* expression level could significantly discriminate between the relapsed and MRD− patients with 69.57% sensitivity and 69.57% specificity. (AUC = 0.7023, *P* = 0.0187). Figures were generated using GraphPad Prism8.0.2 (https://www.graphpad.com/). *MRD−* patients sensitive to chemotherapy, *MRD+ *patients resistant against one year chemotherapy, *Control *non-cancer individuals; *P < 0.05, **P < 0.01.

The relative gene expression levels of *FASN* in MRD+ and MRD− patients are presented in Fig. 2a. A significantly higher mRNA expression level of *FASN* was determined in MRD+ patients compared with the MRD− patients [1.841 ± 0.3311, n = 9 vs. 0.9242 ± 0.1134, n = 31, *P* = 0.0021]. Moreover, ROC curve analysis introduced the mRNA *FASN* level as a prognostic biomarker which may distinguish MRD+ from MRD− ALL patients. The total area under the curve (AUC) was 0.82, confirming the ability and accuracy of this measurement to classify the innate drug resistant patients from the sensitive group (95% CI 0.675–0.978, *P* = 0.0039) (Fig. 2a,b).

In order to determine the association between *FASN* and adaptive drug resistance, the expression levels of *FASN* was measured in 25 relapsed patients. It was revealed that *FASN* was significantly upregulated in ALL relapsed group compared with the MRD− patients (1.169 ± 0.15 vs. 0.7669 ± 0.09448, mean ± SEM, *P* = 0.0180). Moreover, ROC curve analysis revealed that *FASN* expression levels could discriminate between the relapsed and MRD− patients (AUC = 0.7023, 95% CI 0.545–0.891, *P* = 0.0187) (Fig. 2c,d).

### Effect of ginger extract on *FASN* expression in primary ALL cells

**Figure 3:**
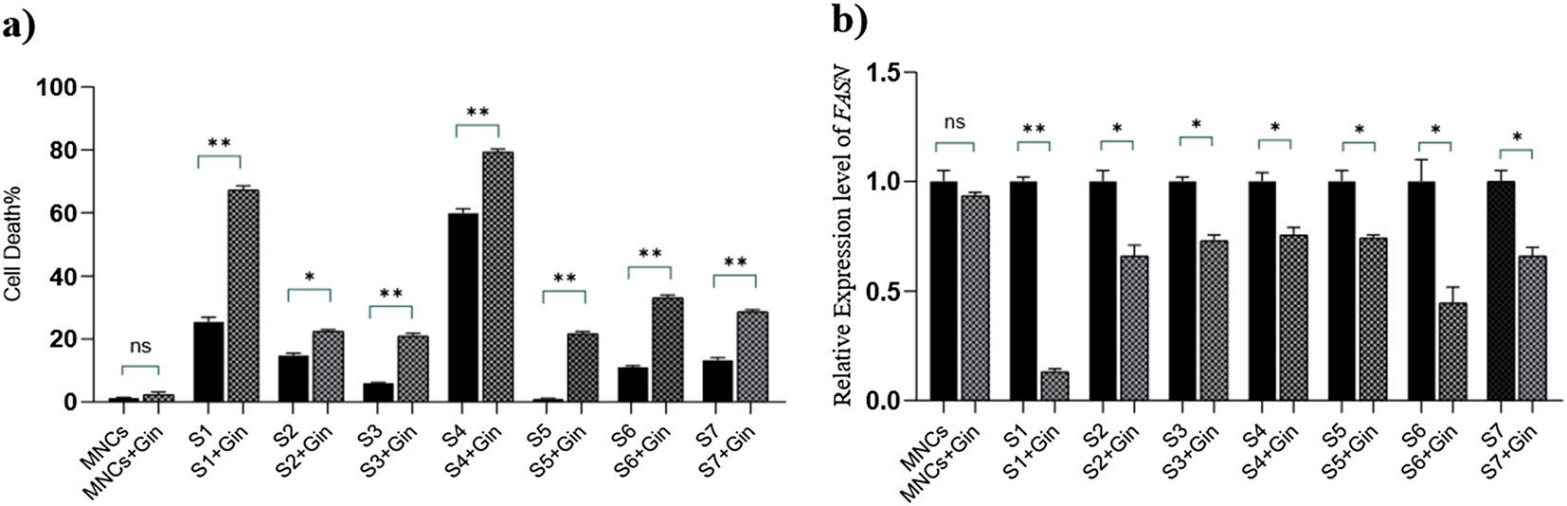
Effect of ginger extract on the ALL relapsed patients primary cells and normal MNCs and its possible correlation with FASN expression levels. (**a**) Malignant cells were isolated from patient bone marrows, at relapse, using Ficoll gradient media. Freshly isolated ALL primary cells and normal MNCs were treated with 167 μg/ml ginger extract for 48 h. Quantification of cell death induction was determined via Annexin V/PI double staining and flow cytometry analysis. Results revealed that cell death increased significantly in malignant cells after ginger treatment (*P* = 0.0023). (**b**) To identify the possible mechanism through which ginger extract may apply its anti-multidrug resistant impact on relapsed samples, real-time quantitative PCR was performed to determine the mRNA expression levels of *FASN* gene. Results showed that the relative amount of *FASN* mRNA expression was decreased significantly in ginger treated samples compared with the untreated specimens (*P* = 0.0031). Figures were created using GraphPad Prism8.0.2 (https://www.graphpad.com/). *MNCs *mononuclear cells, *S *sample, *Gin *ginger, *P < 0.05, **P < 0.01.

The anti-leukemic effect of ginger extract was previously introduced by our group. Moreover, it was shown that this effect was not attributed to the expression levels of ABC transporters^14^. In order to identify the possible mechanism through which ginger could conquer ALL multidrug resistance in patients primary cells, fresh samples were collected from 7 children with relapsed ALL and 1 non-cancer control, treated with 167 μg/ml ginger extract for 48 h, and analyzed for any post-treatment alteration of the *FASN* expression levels. Cell death was measured using Annexin V/PI double staining and flow cytometry analysis. Results supported our previous data considering the significantly increased cell death in ginger treated patient samples compared with the untreated cells [39.11 ± 9.089% vs. 18.80 ± 7.433%, mean ± SEM, *P* = 0.0023]. In addition normal mononuclear cells (MNCs) were not significantly sensitive to proliferation inhibition of ginger extract (Fig. 3a). On the other hand, RT-PCR showed that *FASN* expression in relapsed patients was decreased upon cells exposure to ginger in comparison with the untreated samples (0.5894 ± 0.08593, mean ± SEM, *P* = 0.0031) but in normal MNCs were not significantly decreased (Fig. 3b).

### Effect of ginger extract on CCRF-CEM and dexamethasone resistant CCRF-CEM/MVCD subline

**Figure 4:**
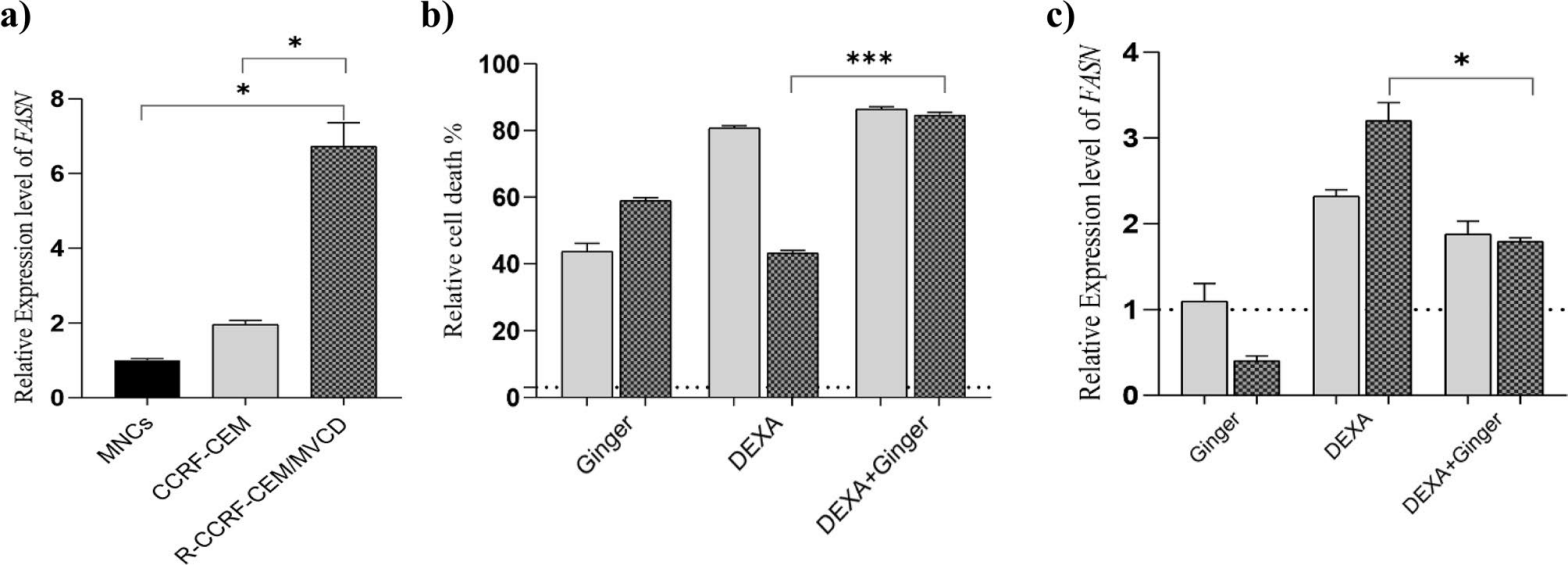
Effect of the ginger extract/dexamethasone combination on multidrug resistant CCRF-CEM/MVCD subline. (**a**) Increased mRNA expression level of *FASN* was observed in the multi-drug resistant R-CCRF-CEM/MVCD subline compared with the normal MNCs and CCRF-CEM parent cell (*P* = 0.0119 and *P* = 0.0241, respectively). mRNA expression levels were normalized by using the expression levels of the housekeeping gene, GAPDH. (**b**) CCRF-CEM and CCRF-CEM/MVCD cells were seeded into 96 microwell plates and incubated with 1,000 μM dexamethasone alone and combined with 167 μg/ml ginger extract for 72 h. Subsequently, cell death was measured using MTT assay. Data showed that the cytotoxic effect of dexamethasone alone was significantly less than that of the dexamethasone/ginger extract combination, (**c**) *FASN* expression levels were assessed using real time PCR. Results showed that *FASN* expression levels were increased in dexamethasone treated cells compared with the untreated cells. Furthermore, *FASN* expression was significantly decreased in dexamethasone/ginger compared with dexamethasone alone treated cells. Figures were created using GraphPad Prism8.0.2 (https://www.graphpad.com/). ( ) CCRF-CEM, ( ) CCRF-CEM/MVCD, ( ) untreated cells, *P < 0.05, **P < 0.01, ***P < 0.001.

The cytotoxic effect of ginger extract on the in house multidrug resistant CCRF-CEM/MVCD subline was previously defined^14^, and RT-PCR showed overexpression of *FASN* in this cell line compared with normal MNCs and its parental cell (*P* = 0.0119 and *P* = 0.0241, respectively) (Fig. 4a). Considering our previous data regarding CCRF-CEM/MVCD, among diverse examined chemotherapy drugs, dexamethasone showed the highest half maximal concentration (IC50) for inhibiting cell growth (Table 2). To investigate the possible impact of ginger in generating CCRF-CEM/MVCD sensitivity to dexamethasone, cells were treated with ginger extract, alone and in combination with dexamethasone. MTT assay was performed in addition to RT-PCR in order to determine the expression levels of *FASN*. Results showed that the cytotoxic effect of dexamethasone/ginger extract was significantly more than that of the dexamethasone alone. In other words, cell viability was reduced down to 15.4 ± 0.821% in the presence of a combination of 1,000 μM dexamethasone with 167 μg/ml ginger, compared with dexamethasone alone (56.794 ± 0.808%, *P* = 0.0008) (Fig. 4b). Subsequently, RT-PCR demonstrated decrease in *FASN* expression followed by combination therapy compared with single drug treatment [1.791 ± 0.043 vs. 3.2 ± 0.210 (mean ± SEM; n = 2), P = 0.0225] (Fig. 4c). Interestingly, *FASN* expression level was increased in response to incubation with dexamethasone alone (*P* = 0.0116).

**Table 2:** IC50 values of the drugs to which R-CCRF-CEM/MVCD cell subline is resistant.

Chemotherapy drugs	CCRF-CEM IC50 (µM)	R-CCRF CEM/MVCD IC50 (µM)
MTX	0.185	225
Ara-C	0.077	4.6
Vincristine	0.00775	0.075
Dexamethasone	576	1,350
Doxorubicin	0.043	0.048

### Docking results of the thioesterase (TE) domain

The molecular surfaces of the FASN TE and KS domains were illustrated using in silico studies (Fig S1). The related interacting residues and binding energy of each docked ligand to the active site of the TE domain were calculated and demonstrated in Table 3. As shown, among diverse ginger phytochemicals (Fig S2), gingerenone family molecules had the highest affinity to the substrate binding site of the TE domain. Interestingly, the binding energies of these molecules were as low as fenofibrate, an experimentally-proved TE inhibitor^15^, and their binding energies were markedly lower than orlistat; which is another known TE inhibitor.

**Table 3:** Docking results of FASN thioesterase (TE) domain with selected phytochemicals. The compounds named in the second column are sorted by their binding affinity to the active site of TE. TE domain inhibitors are colored in bold.

	Ligand	Binding energy (kcal/mol)	Interacting residues	
			H-bonds	Hydrophobic interactions
1	Gingerenone C	− 7.5	Leu2222	Ile2250; Glu2251; Phe2370; Phe2371; Gln2374; Phe2375; Phe2423; Leu2427
2	Gingerenone B	− 7.4	Ser2308; Tyr2343(2)	Ile2250; Tyr2309; Glu2366; Ala2367; Phe2370; Phe2423; Leu2427; Ala2430
3	**Fenofibrate**	− 7.4	Tyr2343	Leu2222; Ile2250; Glu2251; Tyr2309; Phe2370; Gln2374; Phe2423
4	Isogingerenone B	− 7.2	Ser2308; Tyr2309; Tyr2343(2); Glu2431	Ile2250; Ala2363; Glu2366; Ala2367; Phe2370; Phe2423; Leu2427
5	Gingerenone A	− 7.1	Ser2308(2); Tyr2309	Ile2250; Glu2366; Ala2367; Phe2370; Phe2423; Leu2427
6	Alpha-Farnesene	− 7	–	Leu2222; Ile2250; Glu2251; Phe2370; Phe2371; Gln2374; Phe2375; Phe2423; Leu2427
7	**Orlistat**	− 6.7	Ser2308, Tyr2343	Leu2222, Ile2250, Glu2251, Tyr2307, Phe2370, Phe2371, Gln2374, Leu2427, His2481
8	10-Shogaol	− 6.6	–	Leu2222; Ile2250; Glu2251; Glu2366; Ala2367; Phe2370; Phe2371; Gln2374; Phe2375; Phe2423; L2427
9	Beta-bisabolene	− 6.6	–	Leu2222; Ile2250; Glu2251; Phe2370; Phe2371; Gln2374; Phe2375; Phe2423; Leu2427
10	10-Gingerdione	− 6.5	Ser2308; Tyr2343	Ile2250; Tyr2309; Phe2370; Phe2371; Gln2374; Phe2423; Phe2430
11	**Orlistat (hydrolyzed form)**	− 6.5	Tyr2343	Ile2250; Tyr2309; Ala2363; Glu2366; Ala2367; Phe2370; Phe2371; Gln2374; Phe2423; Tyr2424; Leu2427; Glu2431
12	Zingiberene	− 6.4	–	Leu2222; Ile2250; Phe2370; Phe2371; Gln2374; Phe2375; Phe2423
13	Beta-sesquiphellandrene	− 6.4	–	Ile2250; Phe2370; Phe2371; Gln2374; Phe2375; Phe2423
14	10-Gingerol	− 6.4	Ser2308; Tyr2343	Ile2250; Glu2251; Tyr2309; Phe2370; Phe2371; Gln2374; Phe2423; Ala2430
15	Alpha-curcumene	− 6.4	–	Leu2222; Ile2250; Phe2370; Phe2371; Gln2374; Phe2375; Phe2423
16	6-Dehydrogingerdione	− 6.3	–	Ile2250; Try2309; Try2343; Ala2367; Phe2370; Phe2423; Leu2427; Ala2430
17	8-Gingerol	− 6.3	–	Leu2222; Ile2250; Glu2251; Phe2370; Phe2371; Gln2374; Phe2375; Phe2423; Leu2427
18	6-Gingerol	− 6.3	Glu2251, Ser2308(2); Tyr2343; His2481	Leu2222; Ile2250; Phe2370; Phe2371; Gln2374; Phe2375; Phe2423
19	6-Shogaol	− 6.3	Ser2308(2); Tyr2343; His2481	Leu2222; Ile2250; Phe2370; Phe2371; Gln2374; Phe2375; Phe2423
20	Quercetin	− 6.2	Ser2308; His2481(2)	Ile2250; Tyr2309; Tyr2343; Ala2430
21	6-Gingerdiol	− 6.2	Glu2251; Ser2308(2); His2481; Tyr2343	Leu2222; Ile2250; Phe2370; Phe2371; Gln2374; Phe2375; Phe2423
22	6-Paradol	− 6.1	Tyr2343	Leu2222; Ile2250; Glu2251; Phe2370; Phe2371; Gln2374; Phe2375; Phe2423; Leu2427
23	Zingerone	− 5.3	Ser2308	Ile2250; Tyr2309; Tyr2343; Leu2427; Ala2430; Glu2431

**Figure 5:**
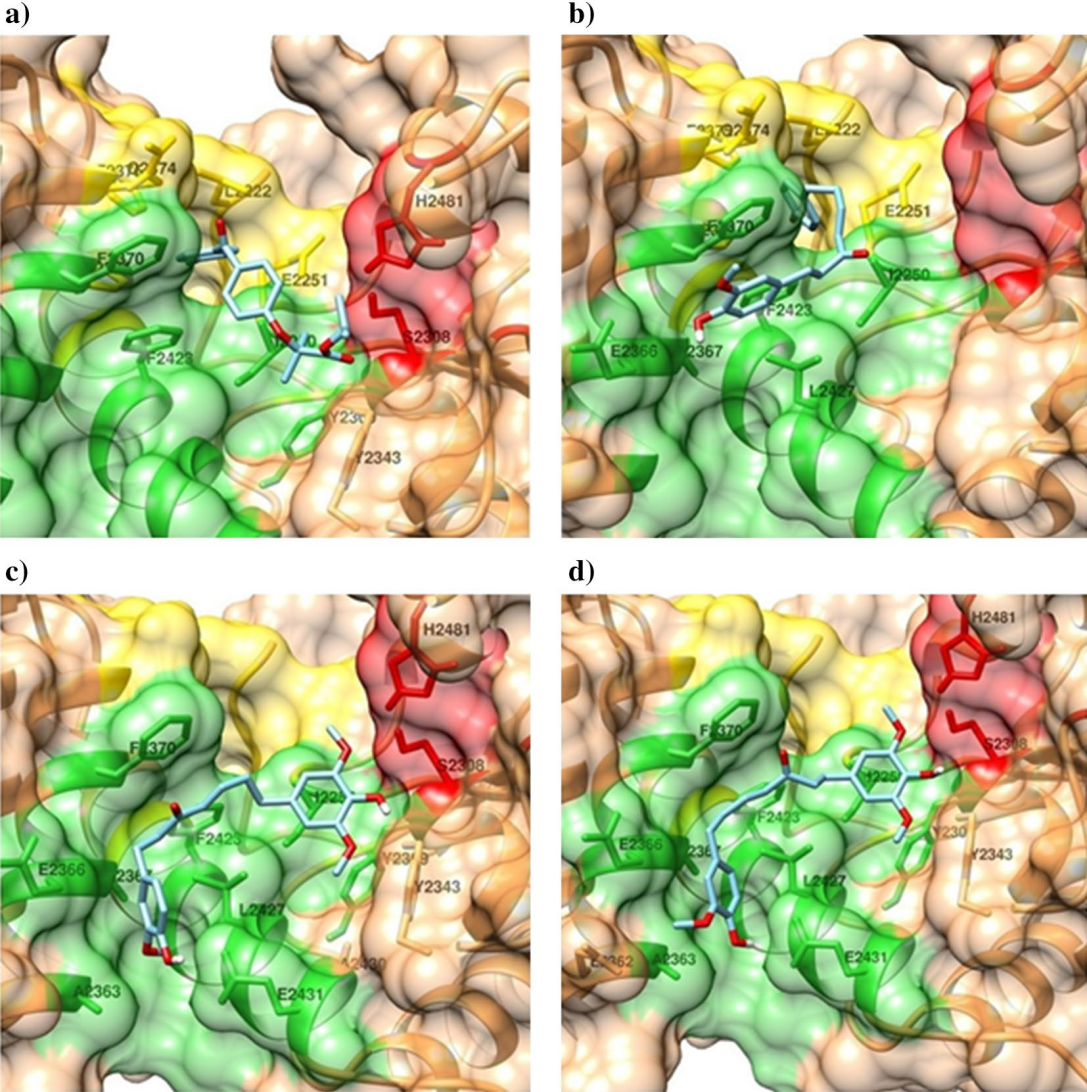
Binding modes of fenofibrate and some gingerenone family members with FASN-TE domain. (**a**) Fenofibrate, (**b**) gingerenone C, (**c**) gingerenone B and (**d**) isogingerenone B. Residues in distance of less than 4 Å of the ligand are labeled and shown in sticks. Red: catalytic triad; green: specificity channel; yellow: interface cavity. Figures were generated using UCSF Chimera 1.13.1^35^.

The interactions of fenofibrate and other gingerenones with the TE domain are illustrated in Fig. 5 and Fig. S3. As shown, gingerenone C covers both, the interface cavity and specificity channel, generating the highest affinity towards the TE domain (see Fig. S4 for more details). Other gingerenone family members, not only lie on the specificity channel and block the substrate binding site, but also extend to the catalytic site and form several hydrogen bonds with important residues for catalytic activity such as Ser2308 and Tyr2343^16^, through which the enzyme could be suppressed.

### Docking results of the ketosynthase (KS) domain

Binding energies of docked ligands to the active-site cavity of the KS domain and interacting residues of each KS-ligand complex are shown in Table 4. In the present study, none of our ligands displayed a significant affinity to the distal substrate binding site. By contrast, several ginger compounds, including quercetin and gingerenone family molecules, showed high affinity to the active-site cavity. Through the occupation of the cavity volume, these ligands may block accessibility to the active site residues Cys161, His293 and His331 and inhibit FASN activity.

**Table 4:** Docking results of FASN ketosynthase (KS) domain with selected inhibitors and phytochemicals. The compounds named in the second column are sorted by their binding affinity to the active site of KS. KS domain inhibitors are colored in bold.

	Ligand	Binding energy (kcal/mol)	Interacting residues	
			H-bonds	Hydrophobic interactions
1	Quercetin	− 8.9	Asp254; Lys257; Asn399	Met205; Pro264; Thr297; Phe393; Phe395; Gly397
2	**Morin**	− 8.5	Asp254; Thr262; Phe393; Phe395; Asn399	Met205; Pro264; Thr297; Gly397
3	Gingerenone B	− 7.7	Thr262; His293; Val299; Phe393	Met205; Tyr222; Phe263; Pro264; Thr295; Thr297; His331; Phe395
4	Gingerenone A	− 7.7	Thr262; His293; Val299; His331	Met205; Tyr222; Phe263; Pro264; Thr297
5	Gingerenone C	− 7.5	Gln259; Th262; His293; Thr297; His331	Met205; Tyr222; Phe263; Val299; Phe395
6	Isogingerenone B	− 7.5	Gln259; Thr262; His293; Thr297; His331	Met205; tyr222; Phe263; Val299; Phe395
7	Beta-sesquiphellandrene	− 7.3	–	Met205; Tyr222; Pro264; His293; Thr297; Val299; Gly300; Phe393; Phe395
8	Zingiberene	− 7.1	–	Cys161; Met205; Tyr222; Pro264; His293; Gly300; Glu304; Phe393; Phe395
9	6-Dehydrogingerdione	− 7	Leu203; Thr262; Thr295	Met205; Phe263; Pro264; His293; Thr297; Gly300; Phe393; Gly394; Phe395
10	Alpha-curcumene	− 6.9	–	Cys161; Met205; Tyr222; Pro264; His293; Gly300; Glu304; Phe393; Phe395
11	6-Gingerol	− 6.9	His331; Phe393	Gly205; Met205; Tyr222; Pro264; His293; Thr297; Gly300; Glu304
12	Beta-bisabolene	− 6.8	–	Met205; Tyr222; Pro264; His293; The297; Val299; Gly300; Phe393; Phe395
13	10-Gingerdione	− 6.8	Thr297; Val299	Met205; Tyr222; Phe263; Pro264; Phe395
14	Zingerone	− 6.6	Thr295; Thr297	Asp254; His264; His293; Gly300; Phe393; Gly394; Phe395; Gly397
15	6-Shogaol	− 6.6	His293; Thr297; His331	Met205; Tyr222; Phe263; Pro264; Val299; Phe395
16	8-Gingerol	− 6.5	Thr262; His293; Thr297; Gly300; His331	Met205; Asn220; Gly221; Tyr222; Pro264; Val299; Phe395
17	10-Gingerol	− 6.5	His293; Thr297; Val299; His331	Met205; Tyr222; Thr262; Phe263; Phe395
18	6-Paradol	− 6.5	His293; Thr297, His331	Met205; Tyr222; Phe263; Val299; Phe395
19	**Cerulenin**	− 6.4	Thr262; Thr297	Met205; Tyr222; Pro264; His293; Thr295; Gly300; Phe393
20	**C75**	− 6.4	His293; Thr295	Met205; Pro264; Thr297; Val299; Gly300
21	6-Gingerdiol	− 6.4	Thr295; Phe393	Met205; Pro264; His293; Thr297; Gly300; Glu304; Phe395
22	10-Shogaol	− 6.3	Thr262; Thr297; Gly300	Cys161; Met205; Tyr222; Phe263; Pro264; His293; Val299; Phe395
23	Alpha-farnesene	− 6.2	–	Met205; Tyr222; Pro264; His293; Thr295; Val299; Gly300; Phe393; Phe395

**Figure 6:**
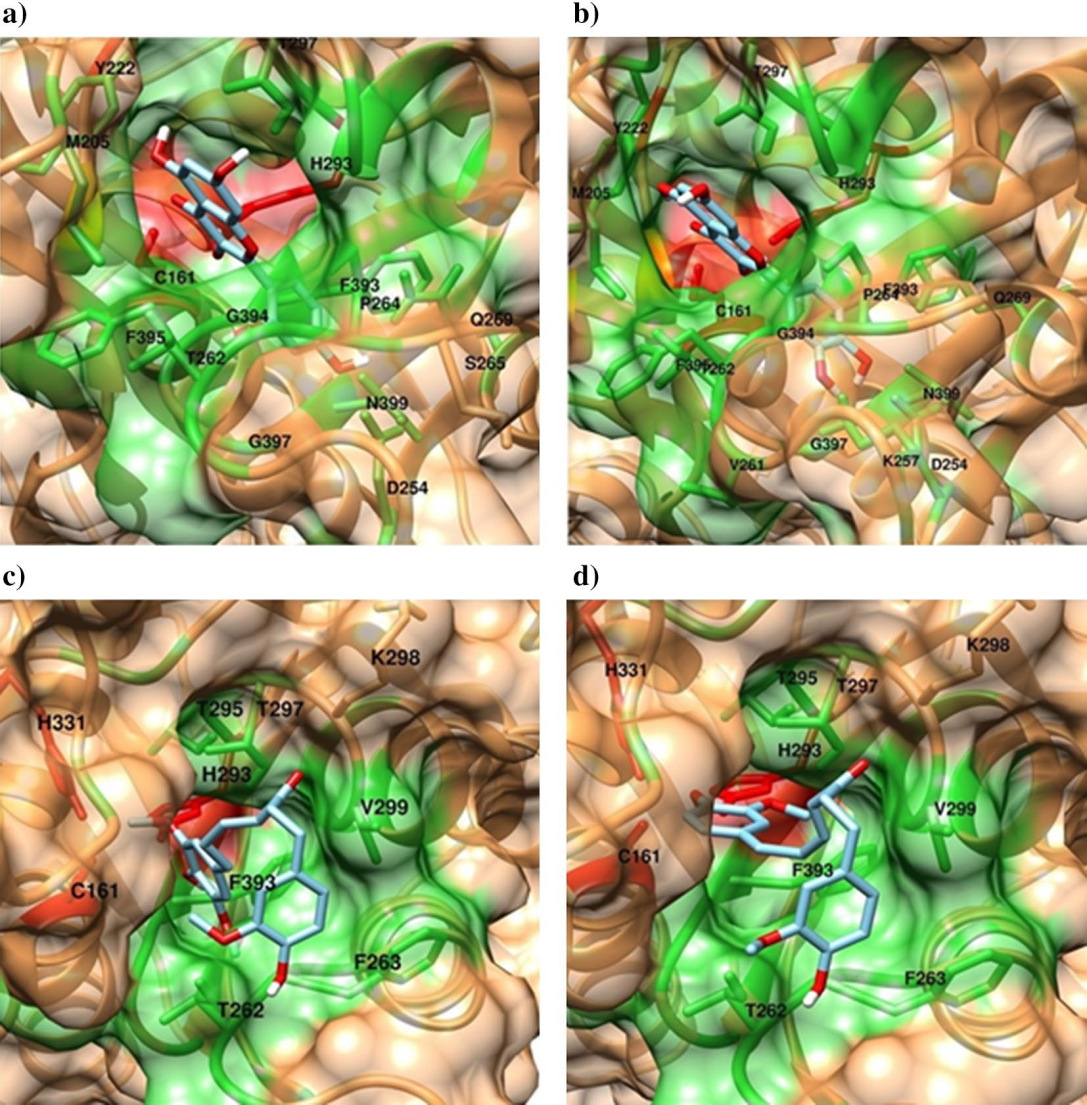
Binding modes of morin, quercetin and two of the gingerenone family molecules with FASN-KS domain. (**a**) Morin; (**b**) quercetin; (**c**) gingerenone B and (**d**) isogingerenone A. Residues in distance of less than 3.5 Å of the ligand are labeled and shown in sticks. Red: active site residues; green: residues lining the active site cavity. Figures were generated using UCSF Chimera 1.13.1^35^.

Morin, the well-known KS domain inhibitor, shares similar molecular scaffold and binding modes to the KS domain with quercetin. However, none of them show direct interaction with the main KS active site residues, implying that their inhibitory function might be due to the blocking of the substrate entry (Fig. 6, Fig. S5). In contrast, the gingerenone family molecules, may not only block the substrate entry, but also enter deep inside the cavity and form several hydrogen bonds with some of the main active site residues such as His293 and His331. (Fig. 6, Figs. S5, S6). Moreover, gingerenones form pi stacking interactions with His293 (data not shown). Cerulenin and C75, the two other docked known inhibitors, showed lower affinity to the KS domain in comparison to the majority of docked ginger phytochemicals.

## Discussion

Despite remarkable advances in the treatment strategies, drug resistance is still a major cause of chemotherapy failure leading to relapse in pediatric acute lymphoblastic leukemia^17^. Multiple studies suggested that metabolic rearrangements, newly recognized as prominent features of cancer^18^, are associated with the development of drug resistance in cancer cells^19^. Changes in lipid metabolism, in particular increased synthesis of fatty acids, are recognized as one of the key hallmarks in several cancer cells. Besides, *FASN* overexpression has shown to be associated with poor prognosis and resistance to chemotherapy^20^.

In the current study, the expression profile of *FASN* was determined in children with ALL. Although our findings showed no increase in *FASN* mRNA levels in de novo ALL patients compared with the control group, *FASN* showed significant upregulation in positive MRD patients known as drug resistant group compared with the drug sensitive or MRD− group. These data supported the hypothesis that *FASN* up-regulation contributes to poor response to chemotherapy. We also examined the potential prognostic value of *FASN* dysregulation in both intrinsic and adaptive drug resistance using ROC curve analysis. Results showed AUCs of 0.82 and 0.7 in discriminating MRD+ from MRD− (*P* = 0.0039) new case samples with one year follow up, and relapsed patients from MRD− individuals (*P* = 0.0187), respectively. Accordingly, it can be hypothesized that *FASN* might be served as a potential prognostic biomarker in pediatric ALL. The increased expression levels of *FASN* in a relapsed patient, compared with the expression levels of this gene at the time of diagnosis (0.075 vs. 1.14, respectively) was another interesting support for this hypothesis (data not shown). Investigating larger populations of ALL paired samples in prospective cohort studies may help intensify the validity of these results.

An increasing number of studies have examined the anti-cancer activity of ginger and its bioactive compounds in drug resistant cancer cells^21^. Our group previously reported the anti-leukemic effect of ginger extract. On the other hand, It was shown that fresh normal peripheral MNCs were not significantly sensitive to proliferation inhibition induced by 50% inhibition concentration of the ginger extract^14^. Furthermore, it was shown that this effect was not ascribed to the expression levels of ABC transporters. To identify the possible targets through which ginger may exert its cytotoxic effect on drug-resistant cells, the expression profile of the *FASN* was analyzed after treating the relapsed ALL patients primary cells with ginger extract. Results revealed that cell death was significantly increased in ginger treated samples compared with the untreated cells. On top of that, *FASN* expression was decreased upon cells exposure to ginger compared with the untreated samples. Our results shared a number of similarities with Impheng et al. findings which demonstrated that [6]-gingerol, one of the derivatives of ginger, reduces de novo fatty acid synthesis, resulting in mitochondrial dysfunction and induction of cell death in HepG2 cells^22^.

Considering the possible contribution of FASN in drug resistance, and the negative impact of glucocorticoids on *FASN* expression levels in B-ALL cell lines^12^, we further investigated the effect of dexamethasone on the sensitive and resistant T-leukemic cells, using RT-PCR followed by MTT assays. The rationale for selecting T cells in these examinations was the aggressive behavior of this phenotype and the low survival rate of T-ALL patients compared with those with B-ALL^23^. Results showed that resistance to dexamethasone was associated with failure to *FASN* downregulation (Fig. 4b). Considering the cytotoxicity of ginger extract on resistant patient primary cells, combination treatments were designed to determine whether ginger extract may overcome resistance to dexamethasone. Intriguingly, results showed that ginger/dexamethasone combined therapy was associated with decreased expression levels of *FASN* and cell growth inhibition (Fig. 4b,c). These data may open up new avenues for improved combination therapies against leukemia drug resistance.

In cancer cells dysregulation of de novo FA synthesis and upregulation of enzymes involved in this pathway occur largely at the transcriptional levels through the activation of sterol regulatory element-binding proteins (SREBPs)^24^. The activity of SREBP is regulated by mTORC1, one of the crucial downstream effector of AKT^25^. In both B-cell and T-cell ALL primary bone marrow samples, AKT hyperactivation has been observed^11^. Similar to this data was our comparison between the transcripts levels of *FASN* in B-ALL and T-ALL patients which revealed no marked difference between these two groups.

Cancer cells are extremely dependent on de novo lipogenesis for proliferation and survival. Therefore, FASN inhibitors seem to play promising role in cancer treatment. It is shown that FASN inhibitors can induce tumor cell apoptosis and sensitize breast cancer cells to chemotherapies^7^. However, off-target activities and detrimental systemic side effects of such components have prevented their clinical development. On the other hand, anticancer activity of the plant components is currently undergoing preclinical evaluation. Therefore, in the next step of this research, molecular docking was used to determine which one of the ginger phytochemicals can interfere with FASN activity. From the six different catalytic domains of FASN, KS (β-ketoacyl synthase) and TE (thioesterase), known as the first and the last catalytic domains of this enzyme, were chosen^26^. Subsequently, we retrieved twenty ginger phytochemicals from published literatures and investigated their inhibitory interactions with FASN.

Since FASN thioesterase domain is involved in palmitate synthesis termination and also in maintaining of the length of fatty acid chain, it is particularly a promising target to inhibit the enzyme activity (TE dom). Orlistat and fenofibrate are the FASN inhibitors which can prevent tumor growth and induce malignant cell death through blocking the TE domain^15^. In order to predict the inhibitory effects of ginger phytochemicals on FASN activity, they were docked with the crystal structure of TE domain and their binding energies were compared to those of orlistat and fenofibrate, in order to prioritize these ligands. Docking results revealed the binding energies of fenofibrate and orlistat to be − 7.4 and − 6.7 kcal/mol, respectively. Of all docked ginger phytochemicals, gingernone family molecules showed the highest binding affinity to FAS-TE domain (C form: − 7.5 kcal/mol, B form: − 7.4 kcal/mol and A form: − 7.1 kcal/mol) even higher than orlistat which is a US FDA-approved and marketed drug for management of obesity, acting through FAS-TE inhibition^26^ (Fig. 5, Fig. S3). The first catalytic domain of FASN is KS^27^. Concerning the KS domain, several inhibitors such as morin, cerulenin and C75 have been reported^28^. The binding free energy of the 20 selected compounds against FAS-KS domain showed the highest binding affinity to the KS in quercetin (− 8.9 kcal/mol), which was even more than morin inhibitor (− 8.5), followed by gingernones (B form: − 7.7 kcal/mol, A form: − 7.7 kcal/mol and C form: − 7.5 kcal/mol) and Isogingernone B (− 7.5 kcal/mol) (Fig. 6), showing much higher binding affinities than the known KS inhibitors, cerulenin and C75 (− 6.5 kcal/mol). Interestingly, it can be noted that gingernones elicit the greater inhibitory effects on both TE and KS domains among all tested ginger compounds. Collectively, these results suggest that ginger may suppress FASN activity and overcome drug resistance through its gingernones. Additional cell-based studies and test tube experiments including dual luciferase (Firefly-Renila) reporter assays are required to confirm the molecular docking data.

In conclusion, our findings emphasize the significance of fatty acid synthesis as a potential target for leukemia treatment. Moreover, ginger constituents are introduced as promising agents able to effectively overcome drug resistance by possibly reducing FASN expression level and inhibiting its activity. Followed by additional FASN overexpression and knockdown studies confirming the causative role of ginger ingredients in FASN downregulation and drug sensitivity, this laboratory investigation could be taken into consideration for the design of animal model studies followed by clinical trials to evaluate the effect of combined treatment of ginger constituents and chemotherapeutic drugs in multidrug resistant leukemia.

## Materials and methods

### In vitro studies

#### Patients and control samples

65 children with ALL and 22 non-cancer controls were included in the present study. Individuals were referred to Sayed-ol-Shohada Hospital, Isfahan, Iran in 2014–2017 for bone marrow evaluation. The project was performed in accordance with the Declaration of Helsinki and permitted by the Ethics Committee of the University of Isfahan (agreement number 94/31540). All Samples of children with ALL and non-cancer controls were collected with full written informed parents’ consents in compliance with the ethical protocols and standards of Sayed-ol-Shohada Hospital. Two to five milliliters of bone marrow heparinized sample was collected from cALL patients and controls and sent on ice to the Cellular and Molecular Biology laboratory of University of Isfahan. Mononuclear cells (MNCs) were isolated by sedimentation on lymphoprep density gradients (Axis Shailed Diagnostics Ltd., Oslo, Norway), according to the manufacturer recommended protocol.

#### Herbal material and chemicals

Extract of ginger dried root (batch number ZSKY20140123) was purchased from Shaanxi Zhengsheng Kangyuan Bio-medical Co., Ltd (Shaanxi, China). Detailed information about this extract is summarized in Table 1a. Dexamethasone was bought from caspiantamin (Rasht, Iran). Dimethyl-sulfoxide (DMSO) was obtained from Cinnagen (Tehran, Iran). Roswell Park Memorial Institute-1640 (RPMI1640), fetal bovine serum (FBS), and penicillin streptomycin (Pen Strep) were purchased from Bioidea (Tehran, Iran). l-Glutamine was from Gibco (Sao Paulo, Brazil) and phosphate buffered saline (PBS) was bought from Sigma-Aldrich (Munich, Germany). 3-(4,5-Dimethylthiazol-2-yl)-2,5-diphenyltetrazolium bromide (MTT) was obtained from Atocel (Graz, Austria). FITC Annexin-V apoptosis detection kit with PI was purchased from BioLegend (London, United Kingdom). TRIzol reagent was from Invitrogen (California, CA) and Ficoll–Hypaque was bought from Inno-train (Kronberg, Germany).

#### Cell lines and patient primary cells

CCRF-CEM (derived from a 4-year-old girl with T-ALL) human cell lines was purchased from Pasteur Institute (Tehran, Iran). Multidrug resistant CCRF-CEM/MVCD subline was generated in-house. Briefly, CCRF-CEM cells sequentially exposed to stepwise concentrations of Methotrexate (MTX) from 5 nM to 1.2 μM. In order to allow cells reaching regular growth rate, cells were kept in the same concentration of MTX for two or three passages. After full growth recovery, the concentration of MTX was increased by twofold each time. Finally, it was revealed that CCRF-CEM/MVCD subline had developed cross-resistance to a number of other chemotherapy drugs including dexamethasone. For further experiments, parental and resistant cell lines were cultured in RPMI1640 containing 10% (v/v) heat-inactivated FBS and 100 μg/ml streptomycin and 1% (v/v) 100 IU/ml penicillin. Freshly collected patient samples were grown in RPMI-1640 supplemented with 20% FBS and 1% l-glutamine.

#### Cell treatment

Cell lines were seeded in 96-well cell culture plates at a density of 15 × 10^4^ cells per well. Cells were suspended in 100 μl supplemented media and treated with 50 μl of freshly made ginger extract (167 μg/ml) for 72 h. Combination treatment was carried out by the addition of 25 μl ginger extract to the same volumes of dexamethasone (1,000 μM), followed by the same incubation time. MTT assay was initiated by the addition of MTT dye to each well. After 3 h incubation at 37 °C, in order to dissolve the formazan crystals in each well, the supernatant was removed and replaced with 100 μl of DMSO. The absorbance was measured at a wavelength of 492 nm using a Stat Fax-2100 microtiter plate reader (Palm City, FL). Cell viability ratio was evaluated as mentioned before^14^.

#### Flow cytometry analysis

Mononuclear cells isolated from relapsed patients samples and normal MNCs were seeded at a density of 25 × 10^4^ cells per well and treated with 167 μg/ml ginger extract for 48 h. At the end of the treatment period, cells were harvested, washed with PBS supplemented by 0.5% FBS, resuspended in 100 μl of cold 1 × Annexin-V-binding buffer after centrifugation, and incubated with 5 μl of FITC conjugated Annexin-V and 10 μl of PI at room temperature for 15 min. The quantitative analysis of cell death was conducted by BD FACSCalibur Flow Cytometer (London, UK). Data was acquired and analyzed using Cell Quest Pro (BD Biosciences, San Jose, CA) and FlowJo softwares (Tree Star Inc., Ashland, OR).

#### RNA extraction and cDNA synthesis

In accordance with the manufacturer’s protocol, total RNA isolation was performed from treated cell lines as well as MNCs of patient and control samples using TRIzol reagent. Extracted RNA was transformed to cDNA in accordance with the instructions provided by PrimeScript RT reagent kit (Takara, Japan) utilizing random hexamers and oligo dT primers. The obtained cDNA was preserved at − 20 °C for further analyses.

#### Real-time PCR analysis

Gene expression assessment was conducted utilizing ExiLENT SYBR Green master mix and Chromo4 system (Bio-Rad, Foster City, CA), according to the manufacturer’s instructions. Data normalization was carried out utilizing glyceraldehyde-3-phosphate dehydrogenase (*GAPDH*) as the internal control gene. qRT-PCR was carried out in duplicate according to the following cycling conditions: 5 min pre-incubation at 95 °C followed by 95 °C denaturation for 20 s, 60 °C annealing for 30 s, and 72 °C product expansion for 30 s. All relative expression levels were evaluated and reported using the 2^−ΔΔCt^ method. The forward and reverse primers sequences for *FASN* were CCGCTTCCGAGATTCCATCCTACGC and GGATGGCAGTCAGGCTCACAAACG; and for *GAPDH *were GCCCCAGCAAGAGCACAAGAGGAAGA and CATGGCAACTGTGAGGAGGGGAGATT, respectively.

#### Response to chemotherapy

ALL patients were treated based on the Australian and New Zealand Children's Cancer Study Group ALL study (https://www.anzctr.org.au/trial_view.aspx?ID=1568). For evaluation of the treatment response, at the end of the first year, the presence of minimal residual disease (MRD) in new patients was assessed utilizing PCR-SSCP (Polymerase chain reaction coupled single-strand conformation polymorphism) analyses for T-cell receptor gamma (TcRγ) and immunoglobulin heavy chain (IgH) gene rearrangements. MRD provides evidence for the presence of post-therapeutic leukemia cells within the bone marrow and more rarely in peripheral blood circulation. Persistent monoclonality, referred to as MRD+, was considered as drug resistance and non-response chemo-treatment. MRD− individuals were appointed as drug sensitive patients.

#### Statistical analysis

SPSS23.0 and GraphPad Prism8.0.2 softwares were used to analyze the data of each experiment statistically. The Kolmogorov–Smirnov normality (KS test) and Shapiro–Wilk tests were applied to evaluate the normality of data distribution. Kruskal–Wallis and Mann–Whitney two tailed tests were carried out to compare the difference of continuous variables between two groups. The statistical significance of differences between two sets of data were estimated using unpaired nonparametric *t* test. Receiver operating characteristic (ROC) curves and the area under the ROC (AUC) were depicted, using GraphPad Prism, to evaluate the specificity and sensitivity of *FASN* as a prognostic biomarker for ALL patients. The greater the area under the curve, the more accurate the test. All data were expressed as mean ± standard error of mean (SEM). Data were results of 2 to 3 independent experiments which were performed in triplicates for cell line analyses, and in duplicates for patient samples. *P* < 0.05 was considered significant, statistically.

### In silico studies

In order to determine the inhibitory impact of ginger phytochemicals on hFASN through in silico studies, various biologically active ginger compounds were selected from literatures^29–32^. Two domains of the enzyme were selected to perform docking simulation study; the ketosynthase (KS) and thioesterase (TE) domains. These domains are particularly important in targeting and inhibiting the FASN activity since the KS domain initiates the fatty acid synthesis cycle^27^ and the TE domain terminates the cycle by hydrolyzing the thioester bond, which results in releasing the 16-carbon fatty acid, palmitate^33^. To determine and prioritize the significance of the docking results of the chosen phytochemicals, some known inhibitors of these two domains were selected and their binding affinities were determined as well^26^.

#### Docking simulation

Methods for selection and preparation of ligands and receptors were described in the supplementary methods. All molecular docking simulations were performed using AutoDock Vina 1.1.2^34^ on a windows platform. Compounds and the protein preparations as well as results analyses were done using UCSF Chimera 1.13.1^35^, AutoDockTools 1.5.6^36^ and LIGPLOT + 2.1^37^.

AutoDock Vina was used to perform semi-flexible docking simulations in which proteins were considered as rigid, while ligands were allowed to be completely rotatable. Since the majority of our ligands were conformationally-flexible with large numbers of rotatable bonds, exhaustiveness was set to 24 for each docking simulation^38^. Other parameters were set to default. Grid boxes were defined for each domain using AutoDockTools. For TE domain, chain B of the pdb file (ID: 2PX6) was used. Subsequently, the Grid box was adjusted around the catalytic triad (Ser2308, Asp2338, His2481), specificity channel and interface cavity, all of which are important for substrate binding as described by John et al.^16^. For KS domain, chain A of the pdb file (ID: 3HHD) was utilized and since it was comprised of KS-MAT didomain, the KS domain was isolated by removing the sequence from Pro410 to Pro824 as described by Pappenberger et al.^39^. The Grid box was then adjusted around the active site residues (Cys161, His293, His331) which are deep inside the active-site cavity and the distal substrate binding sites^40^. Docking simulation was done for each ligand and results were compared and analyzed.

## Supplementary information

Supplementary Information.
