# Publisher Correction: Fatty acid synthase, a novel poor prognostic factor for acute lymphoblastic leukemia which can be targeted by ginger extract

**DOI:** 10.1038/s41598-020-78089-5

**Published:** 2020-11-25

**Authors:** Maryam Ghaeidamini Harouni, Soheila Rahgozar, Somayeh Rahimi Babasheikhali, Arman Safavi, Elaheh Sadat Ghodousi

**Affiliations:** Department of Cell and Molecular Biology and Microbiology, Faculty of Biological Science and Technology, University of Isfahan, 81746-73441 Isfahan, Iran

Correction to: *Scientific Reports* 10.1038/s41598-020-70839-9, published online 21 August 2020

The original version of the Article contained errors in the author names Maryam Ghaeidamini Harouni and Somayeh Rahimi Babasheikhali which were incorrectly given as Maryam Harouni Ghaeidamini and Somayeh Babasheikhali Rahimi.

The Author Contributions section now reads:

“M.G.H. and S.R.B conceived the ideas and performed the experiments. M.G.H. and E.S.G. wrote the article. A.S. and E.S.G. did and wrote in silico studies. S.R. conceived the ideas, designed the study, wrote and reviewed the article.”

These errors have now been corrected in the PDF and HTML versions of the Article.

