# Supplementary material for: Fatty acid synthase, a novel poor prognostic factor for acute lymphoblastic leukemia which can be targeted by ginger extract
Source: Sci Rep. 2020 Aug 21;10:14072. doi: 10.1038/s41598-020-70839-9 (PMC7442786; doi:10.1038/s41598-020-70839-9)
Supplement: Supplementary file 1 — Supplementary Information. [file 41598_2020_70839_MOESM1_ESM.docx]

**Fatty acid synthase, a novel poor prognostic factor for acute lymphoblastic leukemia which can be targeted by ginger extract**

**Maryam Ghaeidamini Harouni^a^, Soheila Rahgozar^a*^, Somayeh Rahimi Babasheikhali^a^, Arman Safavi^a^, Elaheh Sadat Ghodousi^a^**

**Supplementary methods**

**Ligand selection and preparation**

Ginger phytochemicals and some experimentally-proved inhibitors of KS and TE domains of hFASN, as referenced, were selected from literatures, and 3D coordinates of all these compounds were retrieved from PubChem^1^ and ZINC^2^ databases. All hydrogens were added and the structures were converted to pdb format using UCSF Chimera and then AutoDockTools was utilized for merging non-polar hydrogens, adding gasteiger charges and detecting rotatable bonds.

**Receptor preparation**

Since the crystallization of hFASN (UniprotKB entry: P49327)^3^ as a large multifunctional flexible protein is challenging, only isolated domains of the protein have been crystallized.^4^ In the present study, crystal structures of KS domain (PDB ID: 3HHD)^4^ and TE domain (PDB ID: 2PX6)^5^ were obtained from the Protein Data Bank (PDB) [rcsb.org](http://www.rcsb.org/).^6^ UCSF Chimera was used for removing all non-residue atoms including solvent and ligands, adding hydrogens and energy minimization. Then, AutoDockTools was utilized for merging non-polar hydrogens, addition of gasteiger charges to the structures and adjusting grid box parameters required for docking simulations.

**Figure S1.**

**
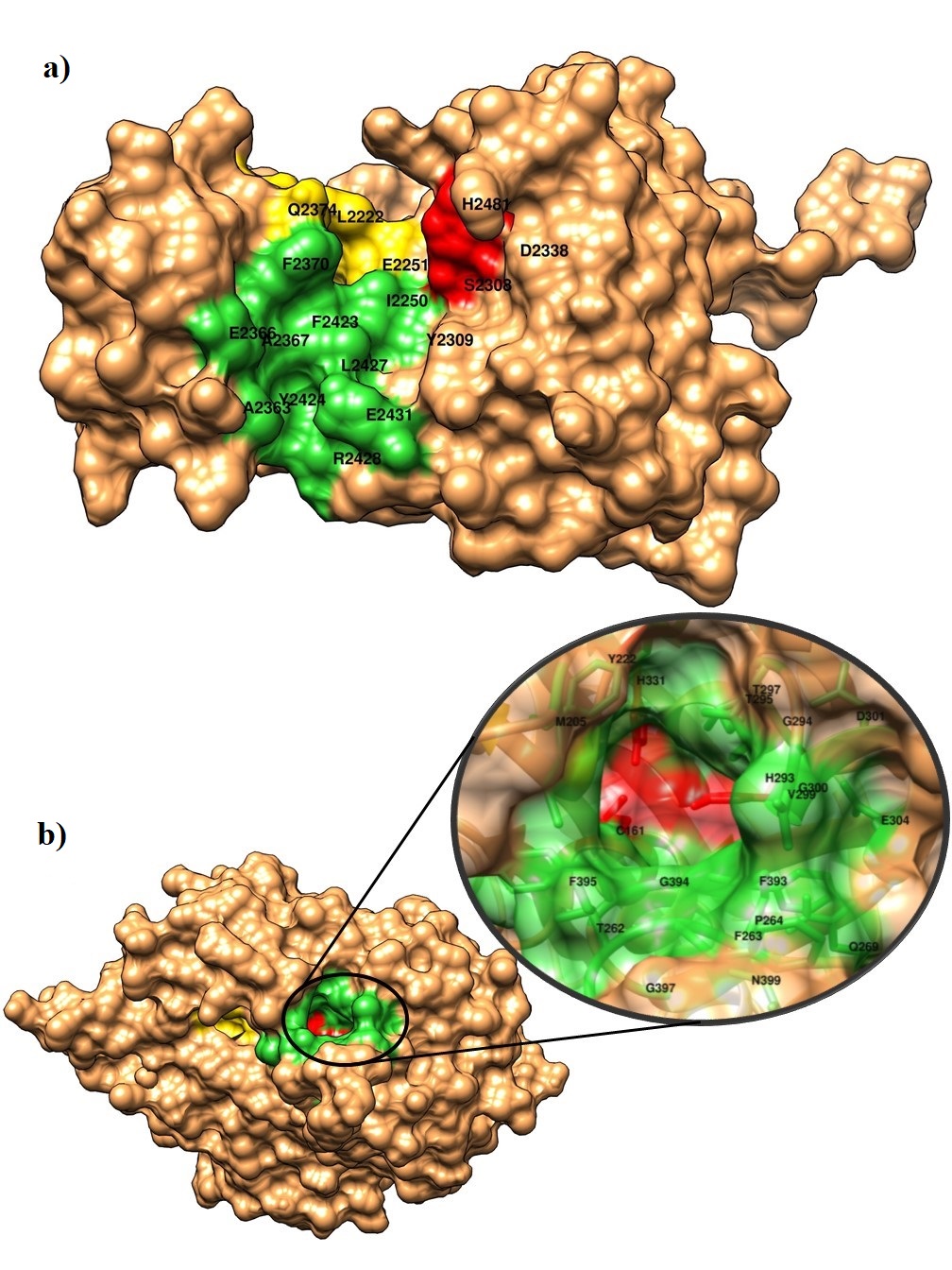
**

**Fig S1. The molecular surfaces of the FASN TE and KS domains.** **a)** Molecular surface of the thioestrase (TE) domain. Red: catalytic triad; Green: specificity channel; Yellow: interface cavity. **b)** Molecular surface of the ketosynthase (KS) domain. Red: active site residues; Green: residues lining the active site cavity; Yellow: distal substrate binding site. Figures were generated using UCSF Chimera 1.13.1.^7^

**
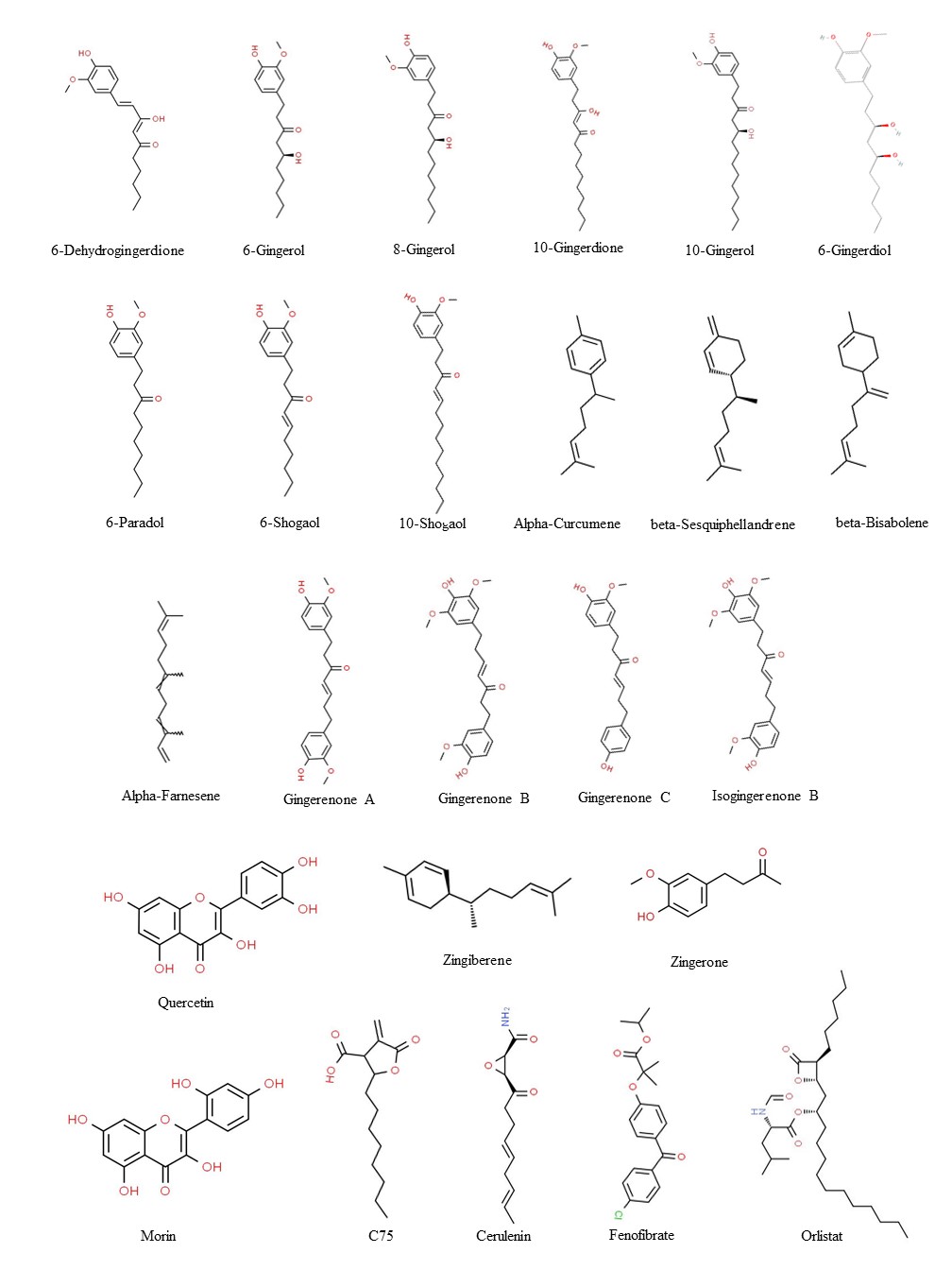
Figure S2.**

**Fig S2**. 2D structures of ginger phytochemicals and inhibitors, obtained from ChemSpider.^8^

**
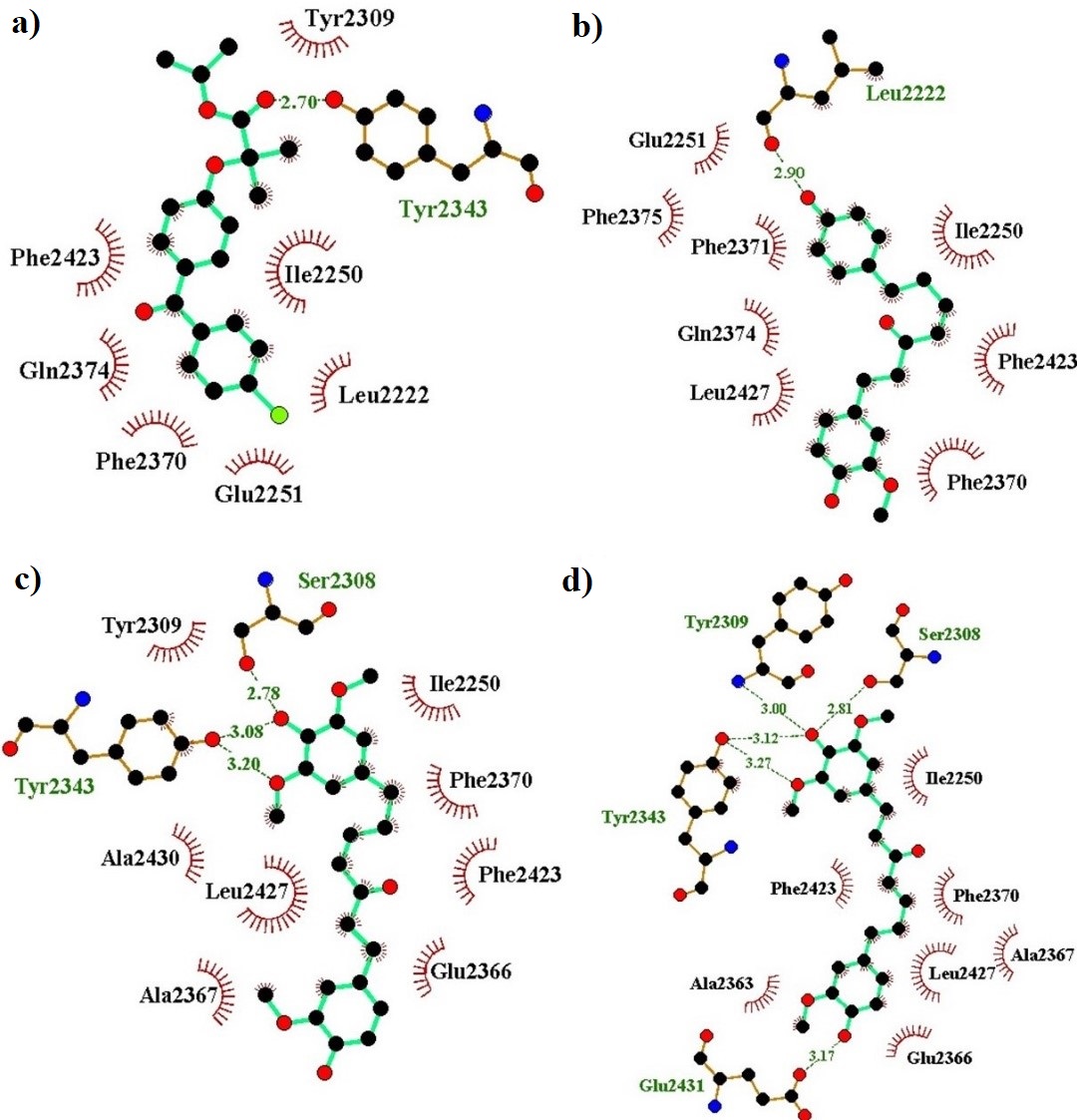
Figure S3**.

**Fig S3.** **The 2D representations of** **fenofibrate and some gingerenone family molecules in complex with FASN-TE domain.** **a)** fenofibrate; **b)** gingernone C; **c)** gingernone B; and **d)** Iso gingernone B. Ligands are displayed in green; Hydrogen bonds are shown in green dashed lines; Residues involved in hydrogen bonds are illustrated as colored spheres. Figures were created using LIGPLOT+ 2.1.^9^

**Figure S4.**

**
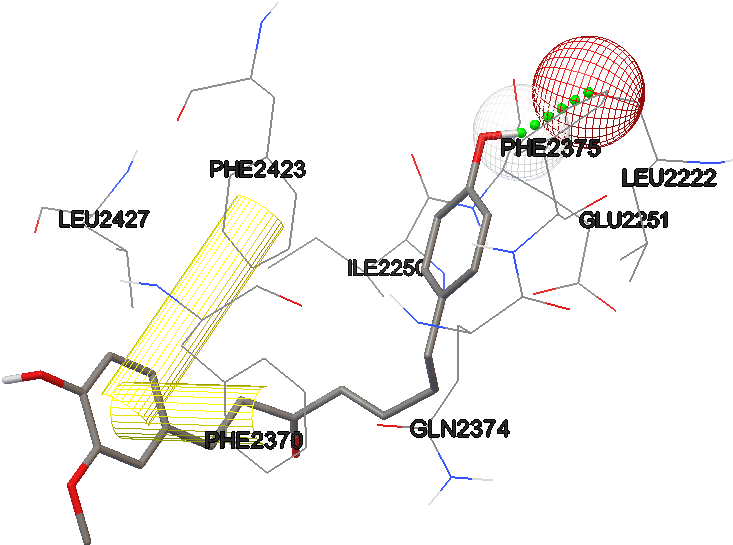
**

**Fig S4. Gingerenone C interactions with the TE domain.** Gingerenone C binds to the TE domain by forming a hydrogen bond with the backbone carbonyl oxygen of Leu2222 and some hydrophobic interactions with residues of interface cavity and specificity channel, especially pi stacking interactions with Phe2370 and Phe2423. Residues in close contact with the ligand are displayed as lines. The hydrogen bond is shown as green spheres and pi stacking interactions are illustrated in yellow cylinders. Figure was generated using AutoDockTools 1.5.6.^10^

**Figure S5.**


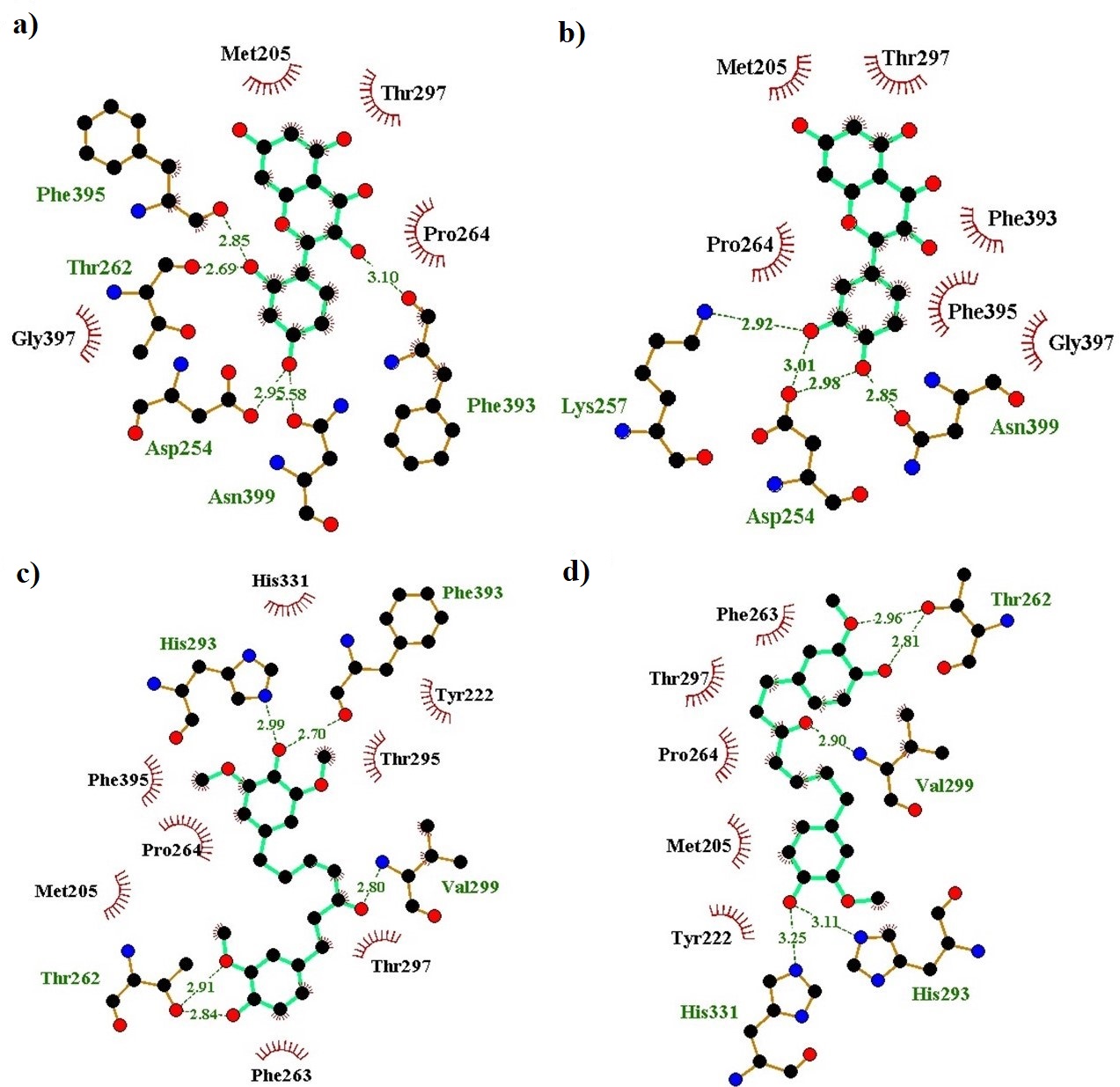


**Fig S5.** **2D illustrations of morin, quercetin, and two of the gingerenone family molecules in complex with the KS domain.** Binding modes of **a)** morin **b)** quercetin, **c)** gingerenone B, and **d)** gingerenone A. Ligands are displayed in green; Hydrogen bonds are shown in green dashed lines; Residues involved in hydrogen bonds are illustrated as colored spheres. Figures were generated using LIGPLOT+ 2.1. ^9^


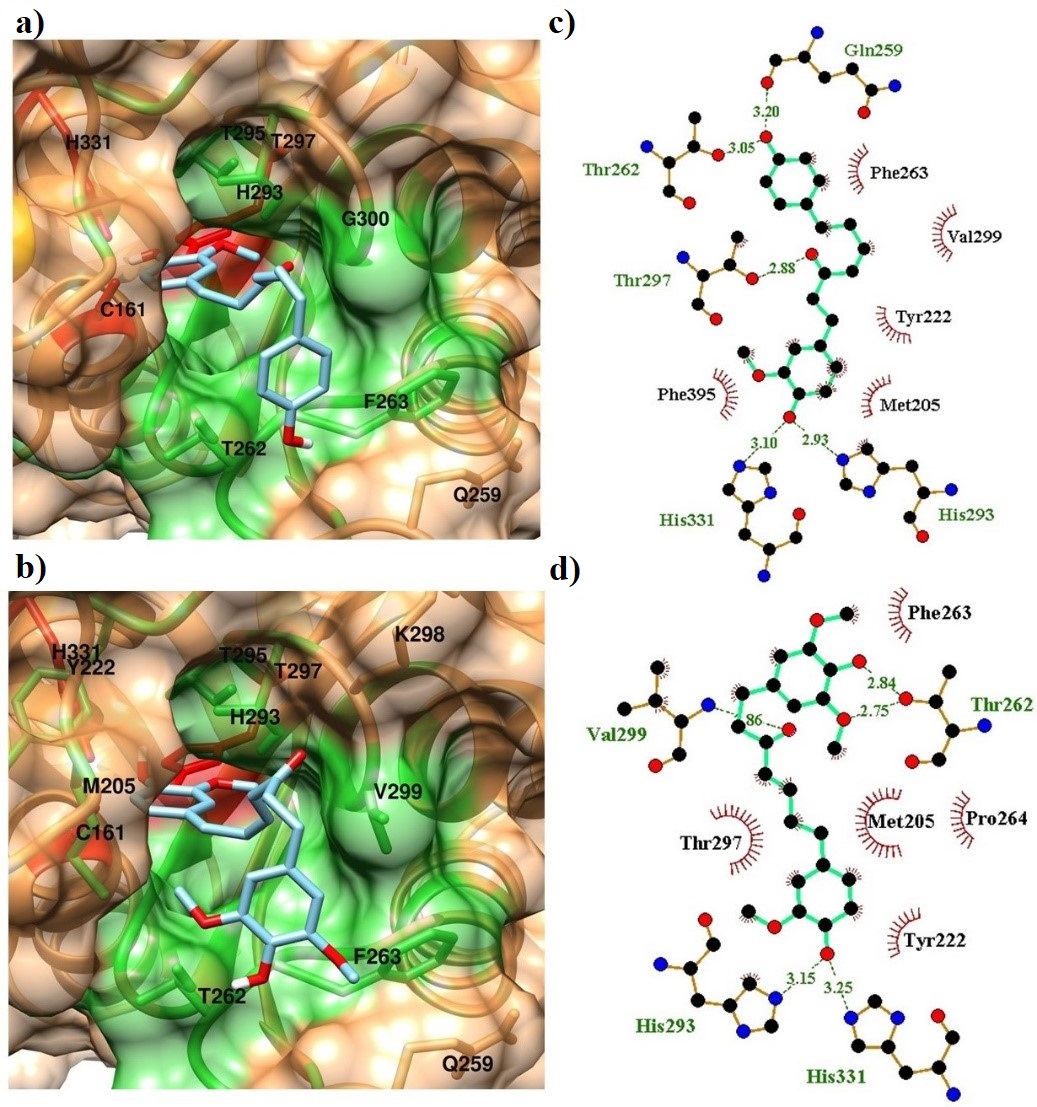
**Figure S6**.

**Fig S6. Binding modes and 2D illustrations of gingerenone C and Isogingerenone B in complex with the KS domain.** Binding modes of **a)** gingerenone C and **b)** Isogingerenone B. Residues in distance of less than 3.5 Å of the ligand are labeled and shown in sticks. Red: active site residues; Green: residues lining the active site cavity. 2D representations of **c)** gingerenone C and **d)** Isogingerenone B. Ligands are displayed in green; Hydrogen bonds are shown in green dashed lines; Residues involved in hydrogen bonds are illustrated as colored spheres. Figures were generated using UCSF Chimera 1.13.1^7^ and LIGPLOT+ 2.1.^9^

1 Kim, S. *et al.* PubChem 2019 update: improved access to chemical data. *Nucleic acids research* **47**, D1102-D1109 (2019).

2 Sterling, T. & Irwin, J. J. ZINC 15–ligand discovery for everyone. *Journal of chemical information and modeling* **55**, 2324-2337 (2015).

3 Consortium, U. UniProt: a worldwide hub of protein knowledge. *Nucleic acids research* **47**, D506-D515 (2019).

4 Pappenberger, G. *et al.* Structure of the human fatty acid synthase KS-MAT didomain as a framework for inhibitor design. *Journal of molecular biology* **397**, 508-519, doi:10.1016/j.jmb.2010.01.066 (2010).

5 John, A., Vetrivel, U., Subramanian, K. & Deepa, P. R. Comparative docking of dual conformations in human fatty acid synthase thioesterase domain reveals potential binding cavity for virtual screening of ligands. *Journal of biomolecular structure & dynamics* **35**, 1350-1366, doi:10.1080/07391102.2016.1184183 (2017).

6 Berman, H. M. *et al.* The Protein Data Bank. *Nucleic Acids Res* **28**, 235-242, doi:10.1093/nar/28.1.235 (2000).

7 Pettersen, E. F. *et al.* UCSF Chimera--a visualization system for exploratory research and analysis. *Journal of computational chemistry* **25**, 1605-1612, doi:10.1002/jcc.20084 (2004).

8 Pence, H. E. & Williams, A. (ACS Publications, 2010).

9 Laskowski, R. A. & Swindells, M. B. (ACS Publications, 2011).

10 Morris, G. M. *et al.* AutoDock4 and AutoDockTools4: Automated docking with selective receptor flexibility. *Journal of computational chemistry* **30**, 2785-2791 (2009).
